# Co-Curricular Activities and Motives for Participating in Physical Activity among Health Sciences Students at Universiti Sains Malaysia, Malaysia

**DOI:** 10.21315/mjms2019.26.1.13

**Published:** 2019-02-28

**Authors:** Garry Kuan, Nurzulaikha Abdullah, Yee Cheng Kueh, Mohd Ismail, Mohd Nazri Shafei, Tony Morris

**Affiliations:** 1 Exercise and Sports Science, School of Health Sciences, Universiti Sains Malaysia, 16150 Kubang Kerian, Kelantan, Malaysia; 2 Unit of Biostatistics and Research Methodology, School of Medical Sciences, Universiti Sains Malaysia, 16150 Kubang Kerian, Kelantan, Malaysia; 3 Department of Community Medicine, School of Medical Sciences, Universiti Sains Malaysia, 16150 Kubang Kerian, Kelantan, Malaysia; 4 Institute for Sport, Exercise and Active Living, College of Sport and Exercise Science, Victoria University, Melbourne, Victoria, Australia

**Keywords:** physical activity, motives, health science, academic, university

## Abstract

**Background:**

The purpose of the study was to compare the motives for participating in physical activity (PA) through the different types of co-curricular activities chosen by health sciences undergraduate students at Universiti Sains Malaysia (USM), Malaysia.

**Methods:**

The participants were university students at USM’s Health Campus, who were invited to volunteer and complete two measures: a demographic form, including the types of co-curricular activities in which the students chose to enrol (sports, uniform and art), and the Physical Activity and Leisure Motivation Scale (PALMS).

**Results:**

A total of 588 university students (female = 79.1%, male = 20.9%) with a mean age of 19.77 (SD = 1.39) participated in the study. The results showed significant differences in the motives of affiliation (*P* < 0.001), appearance (*P* = 0.008) and physical condition (*P* = 0.010) across the types of co-curricular activities in which the students participated. The students who enrolled in sports generally showed higher motives of affiliation, appearance and physical condition for participating in PA than other types of co-curricular activities.

**Conclusion:**

The study findings can provide further insights into the motives for participating in PA among health sciences students and encouragement for students to integrate PA into their daily routines.

## Introduction

Physical activity (PA) and exercise, along with a healthy diet, can bring many important benefits for individuals, including a healthier lifestyle, prolonged life expectancy and higher levels of physical and psychological wellbeing (1, 2). Previous researchers have found that exercise can prolong life expectancy by as much as five years (3, 4). Janssen et al. (1) reported that adults who engage in moderate to vigorous activity for at least 150 min per week can live longer than inactive adults. Promoting the benefits of PA, rather than highlighting the negative consequences of inactivity, then can motivate people to undertake more PA.

Engaging in PA for at least 20 min three days per week also helps increase mental health and reduce stress (1, 2, 4). One factor that can mediate the effect of exercise is socialising with peers, such as spending time with five or more friends for at least two hours per day (1). A study by researchers from the University of Minnesota on 14,800 students at 94 colleges in the United States proved that socialising is an important aspect of participating in PA. In addition, PA enhances mental health and reduces perceived stress. The combination of exercise and socialising, therefore, can have positive impacts on individuals’ health (1).

A popular method of socialising among university students is participating in co-curricular activities. Co-curricular activities are programmes and learning experiences that in some way complement what they are learning in school or university. Such experiences might be relevant to the academic curriculum. Co-curricular activities typically but not always are separate from academic courses (5). They vary by university, although some institutions might have similar activities. The co-curricular activities at Universiti Sains Malaysia (USM), listed in the course registration guidebook, are aimed at emphasising the importance of building soft skills, physical skills and critical thinking to support a sustainable future. The co-curricular activities can be categorised into three main categories: sports (e.g. volleyball, netball and football); independent uniformed groups (e.g. military and police voluntary reserves and university student civil defence); and arts (e.g. guitar playing, photography and drawing). All students must enrol a co-curricular activity for at least a semester during their undergraduate studies.

Co-curricular activities are important for students, and many colleges and universities have made them mandatory. They present an area of institutional activity in which to inculcate values, develop additional life skills, stimulate cooperation among students and provide opportunities for socialising while enjoying student life in beneficial, healthy ways (6, 7). Co-curricular activities also serve as an aspect of university life in which students can gain practical experiences they do not get from classes or lectures, although some co-curricular activities are related to students’ courses of study. Co-curricular activities can help improve study performance (7, 8, 9) by enhancing students’ aspirations to further their education and by reducing absenteeism (6).

The types of curricular activities chosen by students can be related to or contribute to motives for participation in PA. These motives may vary as students choose co-curricular activities based on their preferences. The purpose of this study, therefore, was to determine whether there were any differences in motives for PA participation based on health science students’ co-curricular activities.

## Materials and Methods

### Study Design and Participants

This cross-sectional study involved 590 students from a diverse range of degree programmes related to the medical and health sciences at USM, Kubang Kerian, Kelantan, Malaysia. Convenience sampling was performed to recruit participants, who volunteered to complete the questionnaire. The inclusion criteria were Malaysian undergraduate students who were enrolled in a co-curricular activity during the data collection period and who had strong reading, speaking and writing comprehension skills in Malay. The participants were required to comprehend Malay as the study used a Malay-language questionnaire. After removing problematic responses (i.e. missing data for more than 30% of the questionnaire), data from 588 participants were used in the analysis.

### Demographic Form

The participants were asked to complete a short demographic form collecting information on their gender, age, education, PA participation, PA types, sports experiences and participation in co-curricular activities.

### Physical Activity and Leisure Motivation Scale–Malay (PALMS-M)

The PALMS-M consists of 40 items with eight subscales measuring different types of motives: mastery, enjoyment, psychological condition, physical condition, appearance, affiliation, competition/ego and others’ expectations (10). Each subscale on the PALMS contains five items measured on a 5-point Likert scale, ranging from 1 (strongly disagree) to 5 (strongly agree). Higher scores indicate greater motivation, and vice versa. The PALMS-M has been validated among university students, and study results have indicated that the translated version of the PALMS-M questionnaire was valid and reliable (10). The fit indices from confirmatory factor analysis indicated that the measurement model of the PALMS-M was fit (root mean square error of approximation (RMSEA) = 0.041, standardised root mean square residual (SRMR) = 0.052), and the composite reliabilities for all the subscales ranged from 0.65 to 0.85, indicating that the questionnaire was reliable.

### Procedure

The research was conducted in accordance with the Declaration of Helsinki and approved by the USM Human Research Ethics Committee. In this study, the participants were briefed on and informed about the study, and their informed consent was obtained. The participants completed the demographic form and questionnaire during their co-curricular periods, and the representatives from the group returned them to the university’s co-curricular office.

### Data Analysis

We conducted data entry and statistical analyses using SPSS 22.0. We checked all the demographic forms and PALMS-M questionnaires for any indications of inappropriate responses, such as missing entries, normality and outliers. We used means and standard deviations (SD) to describe the numerical demographic variables and the levels of the participants’ motives for PA participation. We used frequencies and percentages to describe the categorical demographic variables. We conducted one-way ANOVA to compare the differences in motives across the types of co-curricular activities in which the students were involved. If one-way ANOVA indicated significant differences among the groups, pairwise comparisons by Tukey’s post-hoc test were used to identify the pairs in which the differences occurred. Multi-way ANOVA was also conducted adjusting for the possible confounder variable of gender to compare motives between different types of co-curricular activities.

## Results

The majority of the participants were female (79.1%), with a mean age of 19.77 (SD = 1.39). Most were involved in weekly sporting activities, including badminton, football, volleyball, handball, swimming, netball and jogging. The mean level of the motives ranged from the lowest for others’ expectations (3.05; SD = 0.62) to the highest for affiliation (4.21; SD = 0.55). The descriptive statistics for the demographic variables and motive subscales are listed in Table 1.

We conducted one-way ANOVA to test for differences between the means of each motive for PA participation based on the participants’ types of co-curricular activities. The results in Table 2 showed significant differences in the motives of affiliation (*P* < 0.001), appearance (*P* = 0.008) and physical condition (*P* = 0.010) by the types of co-curricular activities selected by the students.

Further analyses of pairwise comparisons between the sports, uniform and art categories of co-curricular activities with significant ANOVA results for the affiliation, appearance and physical condition motivations were performed using Tukey’s post-hoc test (see Table 3). These analyses revealed that the participants who chose sports co-curricular activities reported significantly higher affiliation motives for PA participation than the students participating in the uniform and art categories. Participants in the sports category also reported a significantly higher appearance motive than those in the uniform category and a significantly higher physical condition motive PA than those in the arts category.

After adjusting for the possible confounder variable (gender) in the comparison using multi-way ANOVA, the PA participation motives of affiliation, appearance and physical condition remained significantly different across the comparison groups. Table 4 presents the results of the comparison of PA participation motives by the type of co-curricular activities after adjusting for gender.

## Discussion

The present study yielded interesting findings on differences in the motives for PA participation among university students involved in various types of co-curricular activities. Co-curricular activities were important leisure activities for the students, and the majority participated in various PA in addition to their chosen co-curricular activity. Overall, the average duration of PA found in the present study was 57.34 min per week [SD = 35.89] among the university students. DeLong (12) reported a lower mean of 51.5 min [SD = 27.58] for PA duration among college students. In addition, Ellis et al. (13) reported a mean of 2.8 days of activity per week [SD = 2.2] as the duration of PA of patients clinically diagnosed with mental health conditions. Jaakkola and Washington (14) found that the mean duration of PA was 3.96 [1.71] days per week for an adolescent population. Based on these studies, it is suggested that motivation affects the duration of PA; therefore, more people will engage in exercise for longer durations when their motivation increased (15, 16). The type of co-curricular activity plays a significant role in enhancing motivation for PA participation among university students and young adults. It, therefore, is important to motivate students to get involved in university co-curricular activities that can help increase their overall PA. Many researchers have demonstrated the benefits of PA (17, 18, 19) and the risks of not being involved in any form of PA (4, 20).

Similar results on the direct relationship between motivation and physical activity come from a weight-loss programme studied by Stevens (19). Motives for PA participation were reported to increase the amount of PA in a sample of Finnish adolescents (17), enhance mental health (2) and improve academic performance (2, 9, 21). The present study is novel as it provides insights into the PA participation motives of undergraduate health sciences students.

The descriptive statistics in the present study showed that the eight PALMS motives for participating in PA among USM health sciences students reached average to high levels, with means of 3.05–4.21 on a scale ranging from 1 to 5. This is equivalent to the eight PALMS motives reported by Zach et al. (16) using the Hebrew language version of PALMS, which ranged from 2.65 to 4.45. Using the original English-language version of the PALMS with a Malaysian sample, Molanorouzi (22) also reported that the means of the subscales ranged from 3.66 to 4.19, quite similar to the results of the present study using PALMS-M, the Malay language version. It, therefore, can be concluded that the PALMS survey works in a similar way in Malay as other languages.

The one-way ANOVA results revealed significant differences in the three motive subscales of affiliation, appearance and physical condition based on the type of co-curricular activities chosen by the participants. The students involved in sports co-curricular activities were found to have the highest level of PALMS-M motives, followed by the students who chose uniform activities and then the students who chose arts activities. Regarding the affiliation motive, the sports co-curricular activities showed a significantly higher level than the arts category (*P* = 0.022) and the uniform category (*P* = 0.001). Affiliation has been shown to be a strong motive for participating in team games, whereas art tends to be an individual pursuit, and uniform groups take longer to develop affiliation than sports teams. Regarding the appearance motive, the sports co-curricular activities showed a significantly higher level than the uniform category (*P* = 0.009). This result supports a study (21) suggesting that people who do sports care more about their appearance and seek to become more attractive. Regarding the physical condition motive, the sports co-curricular activities had significantly higher levels than the art category (*P* = 0.016). After adjusting for the possible confounder variable of gender, the results remained the same with significant differences between co-curricular groups with *P*-values of less than 0.050 for affiliation, appearance and physical condition. It correlates to the fact that the sports students engaged in PA during their co-curriculum period in addition to their daily routine. The students who chose sports co-curricular activities, therefore, had higher levels of three motives to participate in PA than the students who opted for the arts and the uniform co-curricular activities.

Academic performance has been proven to be influenced by extra co-curricular activities involvement and to reduce misconduct or indulging in other antisocial behaviours due to the high-stress university student life (23). Darling (21) found that non-athletic students have poorer academic performance, academic aspirations and attitudes towards school. In some cases, participation in non-sports co-curricular activities has a stronger association with being seen as good students and acts as a foundation for further education success (7). Sometimes, bias is possible as only students with good grades opt to participate in co-curricular activities (6). At the same time, longitudinal studies have suggested that sports participation helps raise students’ grades and test scores (6). All undergraduate students should be given opportunities to participate in co-curricular activities according to their preferences. The present study has produced insights for health education providers to share to promote PA participation among university students.

Several limitations of the present study should be addressed in future research. The population of interest was university students, but the present study recruited only students in the health sciences, so the findings cannot be generalised to the whole population of university students. It is suggested that the samples in future research include students from a diverse range of degree programmes at more universities to generalise results to the university student population. In addition, response bias may have occurred during data collection. However, for the present study, all the co-curricular participants available during the data collection period were encouraged to participate in the study and answer honestly, so the bias should be minimised. Other possible confounder variables, such as the type of the participants’ degrees and courses, were not included in the analysis as such data was not collected. Future researchers can address this limitation and capture the types of degrees and courses taken by university students.

## Conclusion

In conclusion, the findings from this study showed that the students who enrolled in sports co-curricular activities generally showed higher motives of affiliation, appearance and physical condition for participating in PA than other types of co-curricular activities. Besides, the findings can provide further insights into the motives for participating in PA among health sciences students and encouragement for students to integrate PA into their daily routines.

## Figures and Tables

**Table 1:** Descriptive statistics for study variables

Variable	Mean (SD)	*n* (%)
Age (year)	19.77 (1.39)	
Gender		
Male		123 (20.9)
Female		465 (79.1)
Race		
Malay		402 (68.4)
Chinese		108 (18.4)
Indian		50 (8.5)
Others		26 (4.4)
Type of co-curricular activity		
Arts		294 (50.0)
Uniform		53 (9.0)
Sports		241 (41.0)
Frequency of exercise per week	2.30 (1.40)	
Duration of exercise per session (minutes)	57.34 (35.89)	
Motives for participating in physical activity:		
Enjoyment	3.93 (.59)	
Mastery	3.55 (.51)	
Competition	3.72 (.66)	
Affiliation	4.21 (.55)	
Psychological	3.79 (.58)	
Physical	3.37 (.45)	
Appearance	3.80 (.51)	
Other’s expectation	3.05 (.62)	

Note: Exercise in this table refers to any planned, structured, and repetitive physical activity that students did during the week (11)

**Table 2:** Comparison of means of motives of PA participation between types of co-curricular activity

Motives of participating in PA	Co-curricular	Mean (SD)	*F*-stat (df)	*P*-value
Enjoyment	Arts	3.91 (0.59)	2.790 (2,579)	0.062
	Uniform	3.78 (0.63)		
	Sports	3.98 (0.57)		
Mastery	Arts	3.55 (0.53)	0.771 (2,578)	0.463
	Uniform	3.47 (0.48)		
	Sports	3.57 (0.51)		
Competition	Arts	3.72 (0.65)	2.336 (2,581)	0.098
	Uniform	3.57 (0.70)		
	Sports	3.78 (0.65)		
Affiliation	Arts	4.17 (0.56)	8.223 (2,584)	< 0.001
	Uniform	3.99 (0.62)		
	Sports	4.30 (0.49)		
Appearance	Arts	3.79 (0.50)	4.833 (2,582)	0.008
	Uniform	3.62 (0.48)		
	Sports	3.85 (0.53)		
Physical	Arts	3.32 (0.48)	4.634 (2,575)	0.010
	Uniform	3.31 (0.32)		
	Sports	3.44 (0.43)		
Psychological	Arts	3.74 (0.62)	1.736 (2,579)	0.177
	Uniform	3.79 (0.53)		
	Sports	3.83 (0.58)		
Other’s expectation	Arts	3.02 (0.65)	1.673 (2,576)	0.189
	Uniform	2.97 (0.44)		
	Sports	3.10 (0.62)		

**Table 3:** Pairwise comparison of mean between type of co-curricular activity for statistically significance motives of PA participation

Motives of participating in PA	Co-curricular	Mean difference (95%	*P-*value
Affiliation	Art versus Uniform	0.17 (−0.026, 0.37)	0.105
	Art versus Sport	−0.13 (−0.24, −0.015)	0.022
	Uniform versus Sport	−0.30 (−0.50, −0.10)	0.001
Appearance	Art versus Uniform	0.18 (−0.012, 0.36)	0.072
	Art versus Sport	0.06 (−0.17, 0.05)	0.369
	Uniform versus Sport	−0.24 (0.05, 0.43)	0.009
Physical	Art versus Uniform	0.01 (−0.15, 0.18)	0.978
	Art versus Sport	−0.11 (−0.30, 0.04)	0.016
	Uniform versus Sport	0.13 (−0.043, 0.30)	0.185

Note: CI = confidence interval

**Table 4:** Comparison of means of motives of PA participation between types of co-curricular activity after adjusting for gender

Motives of participating in PA	Co-curricular	Adjusted mean (95% CI)	*F*-stat(df)	*P*-value
Enjoyment	Arts	3.97 (3.89, 4.05)	2.061 (2, 578)	0.128
	Uniform	3.84 (3.68, 4.00)		
	Sports	4.02 (3.94, 4.09)		
Mastery	Arts	3.62 (3.55, 3.69)	0.595 (2, 577)	0.552
	Uniform	3.53 (3.39, 3.68)		
	Sports	3.61 (3.54, 3.68)		
Competition	Arts	3.79 (3.70, 3.88)	1.747 (2, 580)	0.175
	Uniform	3.64 (3.46, 3.82)		
	Sports	3.83 (3.74, 3.91)		
Affiliation	Arts	4.21 (4.14, 4.28)	7.011 (2, 583)	0.001
	Uniform	4.04 (3.89, 4.19)		
	Sports	4.32 (4.25, 4.40)		
Appearance	Arts	3.82 (3.75, 3.89)	4.409 (2, 581)	0.013
	Uniform	3.64 (3.50, 3.78)		
	Sports	3.87 (3.80, 3.94)		
Physical	Arts	3.38 (3.32, 3.44)	11.894 (2, 574)	0.048
	Uniform	3.36 (3.24, 3.49)		
	Sports	3.47 (3.41, 3.53)		
Psychological	Arts	3.81 (3.73, 3.89)	0.912 (2, 578)	0.402
	Uniform	3.86 (3.70, 4.03)		
	Sports	3.88 (3.80,3.96)		
Other’s expectation	Arts	3.12 (3.04, 3.21)	0.678 (2, 575)	0.508
	Uniform	3.08 (2.91, 3.25)		
	Sports	3.17 (3.09, 3.25)		

Note: CI = confidence interval
